# Oh! Ye of Little Faith

**Published:** 1894-11

**Authors:** C. Carleton Smith

**Affiliations:** Philadelphia, Pa.


### "OH! YE OF LITTLE FAITH."

C. Carleton Smith, M. D., Philadelphia, Pa.

In the August number of The Homœopathic Physician, Dr. Crawford, talking upon the subject of gall-stones, is quoted as saying, in part, before the members of the American Institute, that "he could not be convinced that either Calc-carb., China, or any other drug could be of any value whatever in what he considered a purely mechanical disease, and he would still hold on to Morphine."

Coming from a homœopathic physician, the above seems strange language, especially to the writer of this article, who, having practiced as a Hahnemannian since 1862, has cured such attacks right along by the administration of the similar remedy in each individual sufferer to the total exclusion of Morphia. I do not propose in this paper to argue the matter with the learned doctor. Not only because he says that "he could not be convinced," but also for the better reason that argument is a waste of time in such a mater as this.

Evidently our friend does not exercise implicit faith in the teachings of our master, and hence "follows him afar off." The New Testament teaches us that "faith, even as a grain of mustard-seed, will remove mountains." Then, surely, the same amount of faith exercised by a true follower of Hahnemann ought to remove a little gall-stone, though the "stone be rough and the canal through which it passes be no larger than a goose-quill." In all kindness we ask why it is that the doctor has such supreme faith in Morphia in these cases. It does not cure. Any allopathic physician will admit that. And no one knows it better than the author of the remarks above quoted.

And further than this, the patient who has repeatedly received the narcotic treatment knows that it does not cure. And hence the cry of despair which has come to my ears from these deceived sufferers when for the first time I have responded to their calls, "for Heaven's sake, doctor, don't give me Morphine."

Ah, yes! How many crimes have been committed by the profession in thy name, O Morphia! But come, let us reason together. Is there not a better way of treating these cases than this ancient and musty allopathic method? Experience has taught me that there is—and this better way is "Similia."

By following this gnide I have been enabled to throw aside as being utterly valueless as a guide in practice the mechanical theory of gall-stone colic which has so long blinded the eyes of many of our colleagues to the truth and efficacy of the Hahnemannian law, and thereby cured my patients on purely homœopathic lines.

Theorizing is sometimes good in its way, but when it comes to placing theory alongside of actual bedside practice, it weakens and falls to the ground. In conclusion, I would direct attention simply to two well-known drugs which I have frequently found indicated and, of course, highly efficient in the disease in question, viz.: Lycopodium and Calc-carb. The former is indicated in that class of persons who are of a Lithiatic diathesis, and who have been heavily dosed with tincture of Peruvian bark or its alkaloid, Quinine. They present very sallow faces and suffer from flatulence, this condition causing them to be extremely tender to touch. Must keep their waist-bands loose. When attacked with gall-stone colic, to which such persons as above described are more or less subject, the pain will shoot from region of liver straight across to the left side. When they get up to walk they *bend over*, and also will complain of frequent desire to evacuate bowels, but when they go to the water-closet there is no stool, simply tenesmus.

The Calcarea sufferer is apt to be fat and flabby in his makeup. Has glandular swellings throughout the body. Similar to the Lyc. patient, he cannot bear tight clothing about hypochondria, and pain goes from right to left also, being sharp and most excruciating, causing patient to wring his hands in agony. To distinguish between the two drugs : the Lycopodium patient walks bent over by reason of the great pain, while flatulence is forcibly announced by loud belching, accompanied with greater or less urging to stool at intervals.

The Calcarea patient is made worse by stooping, either in the act or afterward. Sweats profusely, and the pains are greatly relieved by the application of cold, wet cloths, which he begs to have frequently applied.

